# Immortalization of Mesenchymal Stem Cells for Application in Regenerative Medicine and Their Potential Risks of Tumorigenesis

**DOI:** 10.3390/ijms252413562

**Published:** 2024-12-18

**Authors:** Natsuki Yamaguchi, Eri Horio, Jukito Sonoda, Miu Yamagishi, Satomi Miyakawa, Fumihiro Murakami, Hideaki Hasegawa, Yasuhiro Katahira, Izuru Mizoguchi, Yasuyuki Fujii, Daichi Chikazu, Takayuki Yoshimoto

**Affiliations:** 1 Department of Immunoregulation, Institute of Medical Science, Tokyo Medical University, 6-1-1 Shinjuku, Shinjuku-ku, Tokyo 160-8402, Japan; 2 Department of Oral and Maxillofacial Surgery, Tokyo Medical University, 6-7-1 Nishishinjuku, Shinjuku-ku, Tokyo 160-0023, Japan

**Keywords:** immortalization, tumorigenesis, mesenchymal stem cells, cell-based therapy, cell-free therapy, regenerative medicine

## Abstract

Regenerative medicine utilizes stem cells to repair damaged tissues by replacing them with their differentiated cells and activating the body’s inherent regenerative abilities. Mesenchymal stem cells (MSCs) are adult stem cells that possess tissue repair and regenerative capabilities and immunomodulatory properties with a much lower risk of tumorigenicity, making them a focus of numerous clinical trials worldwide. MSCs primarily exert their therapeutic effects through paracrine effects via secreted factors, such as cytokines and exosomes. This has led to increasing interest in cell-free therapy, where only the conditioned medium (also called secretome) from MSC cultures is used for regenerative applications. However, MSCs face certain limitations, including cellular senescence, scarcity, donor heterogeneity, complexity, short survival post-implantation, and regulatory and ethics hurdles. To address these challenges, various types of immortalized MSCs (ImMSCs) capable of indefinite expansion have been developed. These cells offer significant promise and essential tools as a reliable source for both cell-based and cell-free therapies with the aim of translating them into practical medicine. However, the process of immortalization, often involving the transduction of immortalizing genes, poses potential risks of genetic instability and resultant malignant transformation. Cell-free therapy is particularly attractive, as it circumvents the risks of tumorigenicity and ethical concerns associated with live cell therapies. Rigorous safety tests, such as monitoring chromosomal abnormalities, are critical to ensure safety. Technologies like inducible or suicide genes may allow for the controlled proliferation of MSCs and induce apoptosis after their therapeutic task is completed. This review highlights recent advancements in the immortalization of MSCs and the associated risks of tumorigenesis.

## 1. Introduction

**Figure 1:**
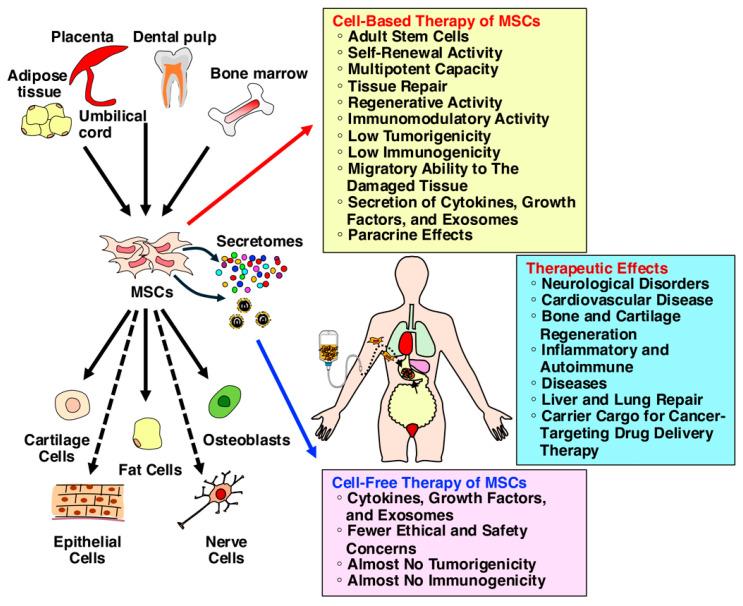
MSCs are adult stem cells characterized by their capacity for self-renewal and multipotency. Due to their immunomodulatory properties, abilities in tissue repair and regeneration, and reduced tumorigenicity, MSCs have demonstrated significant therapeutic potential in treating a wide range of diseases, including bone and neural damage and autoimmune and inflammatory diseases. MSCs release various bioactive factors called secretomes that aid in tissue repair and regeneration. The paracrine effects of MSCs, rather than direct differentiation, play a more critical role in their therapeutic effects. Therefore, the cell-free therapy, using only the secretome from MSCs, can achieve therapeutic benefits mostly similar to MSC-based cell transfer therapy. Cell-free therapy offers several advantages over cell-based therapy. Lower risk of complications, such as embolism, thrombosis, immune rejection, and tumor formation, and fewer regulatory challenges make it a more feasible option for clinical use.

Regenerative medicine aims to repair and restore damaged tissues and organs using stem cells by replacing damaged cells with their differentiated counterparts and by activating the body’s inherent regenerative abilities. Stem cells possess the key traits of self-renewal and multipotency, allowing them to differentiate into a variety of somatic cells. There are different types of stem cells, including pluripotent stem cells (PSCs), such as embryonic stem cells (ESCs), and induced pluripotent stem cells (iPSCs), as well as adult stem cells, such as mesenchymal stem cells (MSCs), hematopoietic stem cells, and neural stem cells (NSCs). MSCs are adult stem cells found throughout the body, characterized by their capacity for self-renewal and multipotency, allowing them to differentiate into various mesenchymal lineages, such as osteoblasts, adipocytes, and chondrocytes (Figure 1) [1,2,3]. While pluripotent stem cells hold great therapeutic potential, they also come with the inherent risk of teratoma formation if any undifferentiated cells remain in the body [4,5]. In contrast, MSCs have demonstrated significant therapeutic effects in treating diseases such as bone and neural damages, as well as autoimmune and inflammatory diseases, due to their immunomodulatory properties and abilities in tissue repair and regeneration [6,7,8]. MSCs present lower risks, including reduced tumorigenicity, which makes them a focus of ongoing clinical trials worldwide [9,10,11]. Generally, two primary mechanisms of action explain the therapeutic potential of MSC-based cell transfer therapies: homing and differentiation and paracrine effects [6,12]. MSCs have a remarkable ability to home in on damaged tissues and organs. Once administered, they migrate to the injury site, where they differentiate into the required cell types, replacing damaged cells and promoting tissue regeneration. MSCs also release various bioactive factors called secretomes, which is the set of molecules secreted from cells into the extracellular space, including cytokines, growth factors, and exosomes, and aid in tissue repair and regeneration [13,14,15]. These factors act on damaged cells and surrounding cells, stimulating their regenerative responses in a process known as paracrine effects.

Recent studies have shown that the half-life of MSCs administered in the body is relatively short, and only a small percentage of administered MSCs remain detectable [16,17]. This suggests that the paracrine effects of MSCs, rather than direct differentiation, play a more critical role in their therapeutic effects. In line with this understanding, research has demonstrated that cell-free therapies, using only the secretome that is the conditioned medium from MSCs, can achieve therapeutic benefits mostly similar to MSC-based cell transfer therapies [18,19]. Cell-free therapy offers several advantages over cell-based therapy. Lower risk of complications, such as embolism, thrombosis, immune rejection, and tumor formation, and fewer regulatory challenges make it a more feasible option for clinical use [18]. Thus, the emerging use of the secretome of MSC represents a promising, safer alternative to direct cell therapy for regenerative medicine applications.

**Figure 2:**
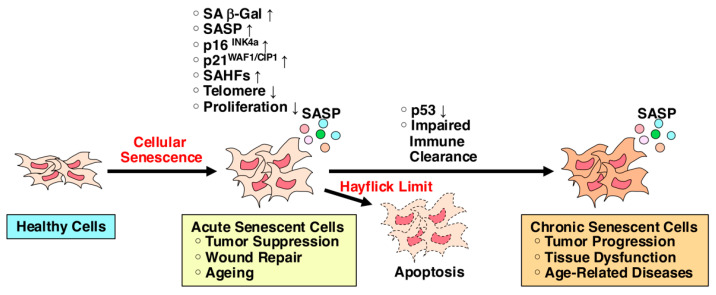
MSCs have a limited capacity for division due to cellular senescence, known as the Hayflick limit. Cellular senescence is a biological mechanism where cells permanently stop dividing but remain metabolically active. It serves as a powerful tumor suppressor mechanism, halting the proliferation of damaged or potentially malignant cells. However, the accumulation of senescent cells over time can also contribute to aging and chronic diseases. Senescence-associated secretory phenotype (SASP) is a phenotype associated with senescent cells, secreting high levels of inflammatory cytokines, and immune modulators and eventually leading to promotion of tumor progression. SA-β-gal, senescence-associated β-galactosidase; SAHF, senescence-associated heterochromatin foci.

**Figure 3:**
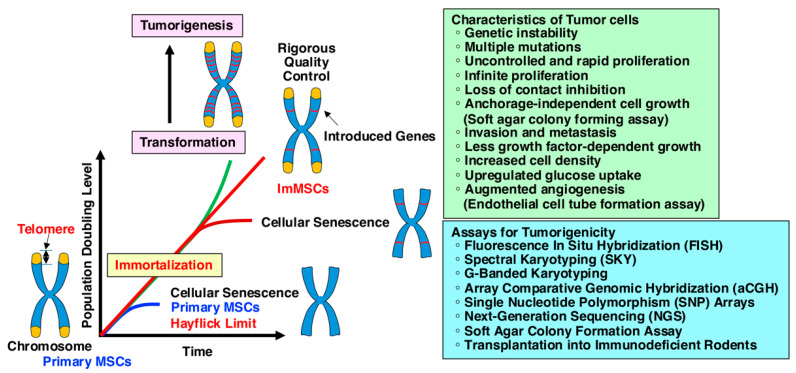
Population doubling level is the total number of times that the cells in a given population have doubled during in vitro culture. As the population doubling level increases, primary MSCs change their phenotype and lose their ability to proliferate, differentiate, and modulate the immune response. Immortalization endows MSCs with indefinite growth, but also may increase the potential risk of genetic mutations and resultant malignant transformation due to prolonged cultivation depending on the culture conditions. Rigorous safety tests, such as soft agar assays, transplantation into immunodeficient rodents, and monitoring for chromosomal abnormalities using FISH or karyotyping, are critical to ensure safety.

ESCs and iPSCs exhibit infinite growth potential, whereas MSCs have a limited capacity for division due to cellular senescence, known as the Hayflick limit (Figure 2) [20]. To enhance the versatility of MSCs and create infinitely expanding MSCs, scientists have developed various types of immortalized MSCs (ImMSCs), which were established by introducing immortalizing genes, such as human telomerase reverse transcriptase (hTERT) and oncogenes [21,22]. ImMSCs have proven to be valuable cell sources for both cell-based therapy and cell-free therapy. ImMSCs are increasingly used in preclinical medicine because, unlike primary cells, they can produce large quantities of cells and be used indefinitely. They also eliminate the issue of donor heterogeneity, which can cause variability in the quality of MSCs from different sources. However, the process of immortalization, typically involving the introduction of immortalizing genes, carries a potential risk of genetic mutations and resultant malignant transformation due to prolonged cultivation depending on the culture conditions (Figure 3) [23,24].

In this review, we explore the recent advancements in the immortalization of MSCs and discuss the associated potential risks of tumorigenesis. By examining the benefits and challenges of using ImMSCs, we highlight the practical necessities of ImMSCs as a reliable cell source for both cell-based therapy and cell-free therapy, with the aim of translating them into practical medicine.

## 2. Hayflick Limit and Telomeres

MSCs can be expanded under in vitro culture and passaged but only several times due to the Hayflick limit (Figure 2) [20]. The Hayflick limit is a concept in cellular biology that describes the number of times a normal human cell population can divide before it stops, due to a process called cellular senescence. It was first discovered by Leonard Hayflick in 1961 [25] and has since become an important principle in the study of aging, cancer, and cellular biology. Cellular senescence is a biological mechanism where cells permanently stop dividing but remain metabolically active [26,27,28]. It serves as a powerful tumor suppressor mechanism, halting the proliferation of damaged or potentially malignant cells. However, the accumulation of senescent cells over time can also contribute to aging and chronic diseases. Senescence-associated secretory phenotype (SASP) is a phenotype associated with senescent cells, secreting high levels of inflammatory cytokines, and immune modulators and eventually leading to promotion of tumor progression [28].

**Figure 4:**
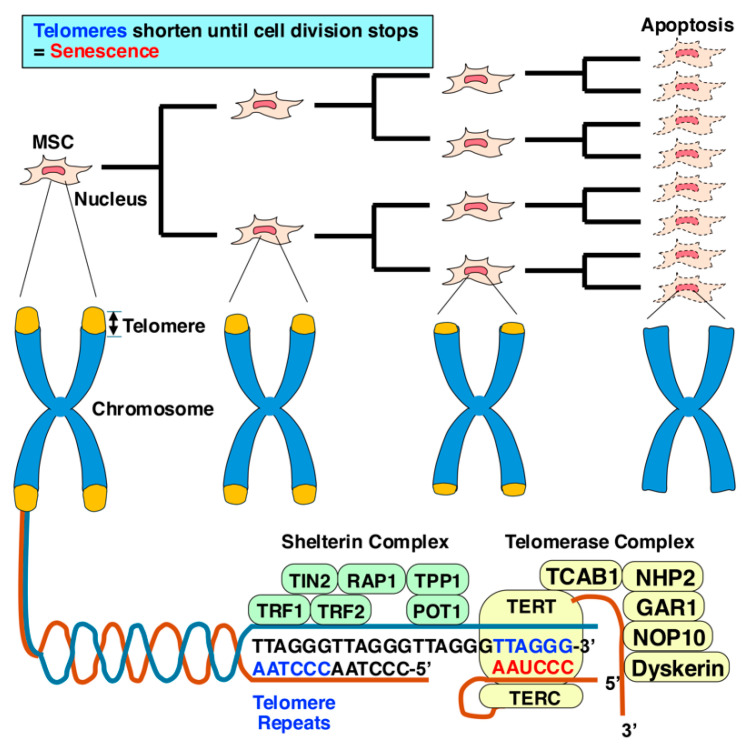
Telomerase is a ribonucleoprotein enzyme essential for synthesizing telomeric DNA, thereby extending the specific repetitive sequences at the ends of chromosomes, known as telomeres. It is composed of two main components: hTERT and hTERC. In cells where hTERT is highly expressed, telomerase can maintain or even increase telomere lengths, preventing cellular senescence.

Humans have 23 pairs of chromosomes, which consist of tightly coiled DNA wrapped around proteins and are located in the nucleus of every cell. Each chromosome has a unique structure, comprising two short arms (the p arms) and two long arms (the q arms), joined together at the center by the centromere. This organization helps to keep genetic information securely housed within the nucleus. Crucially, chromosomes are capped at their ends by regions of repetitive DNA known as telomeres (Figure 4) [29,30,31]. These telomeres play an essential role during cell division, protecting chromosome ends from becoming tangled or frayed, and ensuring accurate DNA replication. Each time a cell divides, the telomeres shorten slightly; eventually, they become so short that the cell can no longer divide, leading to cellular senescence and cell death.

Telomerases, a subgroup of RNA-dependent polymerases, function to lengthen telomeres by adding nucleotide sequences to the ends of chromosomes (Figure 4) [32,33]. This activity allows senescent cells that would typically become postmitotic and undergo apoptosis to surpass the Hayflick limit [34] and potentially achieve immortality, a characteristic often seen in cancerous cells. The catalytic subunit of telomerase, TERT, forms the active component of the telomerase complex along with the telomerase RNA component (TERC) [33]. TERT is responsible for catalyzing the addition of nucleotides, specifically the repetitive TTAGGG sequence, to the ends of telomeres. This process protects the chromosome ends from degradation during multiple rounds of replication. Telomerase activity is typically absent in most normal somatic cells but is present in cancer cells, which often express TERT at high levels. This allows tumor cells to divide and proliferate indefinitely, similar to immortalized cells, contributing to cancer progression.

## 3. Immortalization

**Figure 5:**
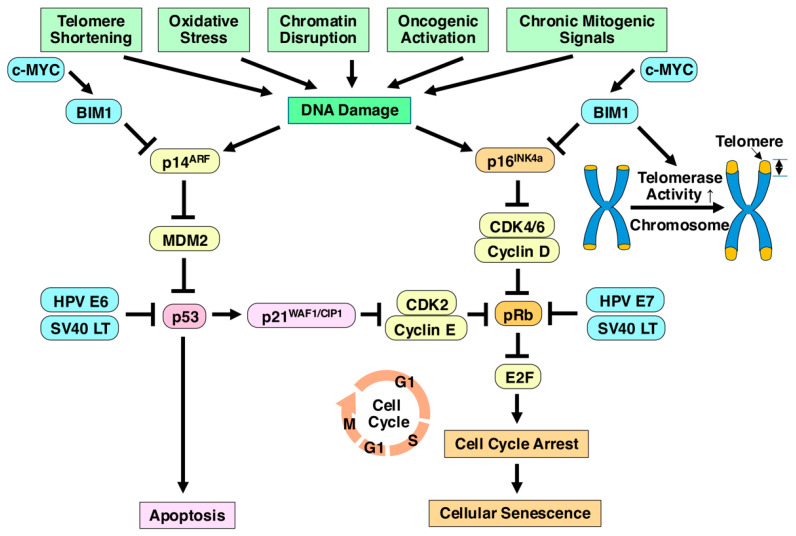
Several target genes were used to achieve cell immortalization through genetic manipulation. TERT, an enzyme that extends telomeres, thus bypassing the Hayflick limit and enabling continuous cell division. c-MYC oncogene promotes cell proliferation and extends the lifespan of cells. Viral oncoproteins from viruses like SV40 and HPV E6 and E7 deactivate crucial tumor-suppressor proteins such as pRb, p53, p16^INK4a^, and p21^WAF1/CIP1^, which are vital for inducing senescence, controlling cell division, and apoptosis.

Cells cultured in vitro have a limited capacity to divide due to the Hayflick limit [20,25,34], and as telomeres shorten with each division, cells eventually stop dividing (Figure 4). To overcome this, one approach is the introduction of TERT, an enzyme that extends telomeres, thus bypassing the Hayflick limit and enabling continuous cell division [33]. Several techniques have been developed to achieve cell immortalization through genetic manipulation (Figure 5, Table 1) [22,35,36]. The most studied methods include the introduction of hTERT, viral oncoproteins, and the c-MYC oncogene. hTERT sustains telomere length and allows for continuous division. The c-MYC oncogene promotes cell proliferation and extends the lifespan of cells. Viral oncoproteins from viruses like simian virus 40 (SV40) and human papillomavirus (HPV) E6 and E7 deactivate crucial tumor-suppressor proteins such as p53, retinoblastoma protein (pRb), p16^INK4a^, and p21^WAF1/CIP1^, which are vital for inducing senescence, controlling cell division, and apoptosis. Deactivating these suppressors increases telomerase activity and aids in immortalization, but this strategy poses risks. The loss of p53 function leads to genomic instability, resulting in errors in DNA replication, polyploidy, and altered chromosome numbers, all of which are associated with cancerous transformations. The loss of p53 is observed in around 50% of human cancers, underscoring the risks of tumorigenesis with these methods [37].

### 3.1. Immortalizing Genes

#### 3.1.1. hTERT Gene Expression

Telomerase is a ribonucleoprotein enzyme essential for synthesizing telomeric DNA, thereby extending the specific repetitive sequences at the ends of chromosomes, known as telomeres (Figure 4) [38]. It is composed of two main components: hTERT and hTERC. In cells where hTERT is highly expressed, telomerase can maintain or even increase telomere lengths, preventing cellular senescence [39]. This is particularly important in contexts where continuous cell division is required, such as in stem cells and certain cancer cells. In most adult somatic cells, telomerase activity is minimal due to tightly regulated expression of hTERT. Immortalization of MSCs using hTERT is one of the most common and efficient methods for extending the lifespan of MSCs [40], enabling them to proliferate beyond their natural senescence limit [41]. Introducing the hTERT gene extends the lifespan of MSCs by maintaining telomere length, preventing replicative senescence. This method has been widely used due to its relative simplicity and effectiveness in immortalizing MSCs without significantly altering their biological properties [42]. hTERT restores telomerase activity in MSCs, preventing telomere shortening and allowing for continuous cell division, bypassing the Hayflick limit, the point at which normal cells stop dividing due to telomere attrition. Cells that overexpress hTERT do not exhibit the typical signs of senescence, such as upregulation of p16^INK4a^ and p53 pathways, and can proliferate beyond the normal limit of 40–60 population doublings [43]. MSCs immortalized by hTERT typically retain their multipotency and differentiation potential, allowing them to continue functioning as stem cells while also having enhanced proliferative capacity [23]. Unlike other immortalization methods using viral oncogenes, hTERT immortalization preserves the normal karyotype, multipotency, and differentiation ability of MSCs [44]. hTERT immortalization alone generally does not induce oncogenic transformation, making it safer compared to methods involving viral oncogenes, such as SV40 Large T antigen (LT) [45]. While hTERT itself is less likely to induce tumorigenicity, prolonged culture of immortalized cells increases the risk of acquiring mutations that could lead to cancer depending on the culture conditions [46]. hTERT-ImMSCs can be expanded in large quantities for potential use in cell-based therapy, as they retain their ability to differentiate into bone, cartilage, and other mesenchymal lineages.

**Table 1:** Pros and cons of immortalization techniques for MSCs.

Immortalization Techniques	Pros	Cons	References
hTERT	◦ Maintains telomere length, extending replicative lifespan ◦ Retains stem cell properties ◦ Reduced risk of spontaneous mutations compared to other methods	◦ Potential oncogenic risk due to TERT activation ◦ Requires thorough safety evaluations	[41,42,43,44,45,46]
SV40	◦ Efficient and rapid immortalization ◦ Inactivates p53 and pRb pathways ◦ Commonly used and well-documented in cell research	◦ High risk of tumorigenicity due to disruption of p53 and pRb pathways ◦ Alters cellular physiology	[47,48,49,50]
HPV E6 and E7	◦ Maintains cell proliferation by inactivating p53 and pRb pathways ◦ Relatively simple transduction method	◦ Significant oncogenic risk ◦ Alters native MSC functionality	[51,52,53,54]
BMI1	◦ A member of the polycomb group of proteins, cooperating with c-MYC and promoting tumorigenesis ◦ Inhibits the expression of p16^INK4a^, thereby blocking the senescence pathway ◦ Enhances the expression of hTERT, maintaining telomere length and enabling cells to divide indefinitely	◦ Less efficacy in cell immortalization	[55,56,57,58]
c-MYC	◦ Promoting DNA damage through its regulation of various genes, which lead to genomic instability ◦ Promotes the overexpression of hTERT, thereby contributing to cellular immortality	◦ Potent oncogene promoting cancer development and progression	[59,60,61]
CRISPR/Cas9	◦ High specificity for targeted immortalization ◦ Reduced off-target effects compared to viral methods ◦ Allows multi-gene modifications	◦ Requires advanced expertise and infrastructure ◦ Potential for unforeseen genomic instability	[62,63,64,65]
tsA58(SV40 LT mutant)	◦ Temperature-sensitive mutations (active at 33 °C, inactive at 39 °C) ◦ Reduced risk of permanent transformation	◦ Requires precise control of culture conditions ◦ Limited application in large-scale production	[66]
c-MYCER^TAM^	◦ Reversible, safer, and more controllable immortalization ◦ A fusion protein of a human c-MYC and a modified mouse estradiol receptor (ER) ◦ Addition of 4-OH binds to the ER, activating c-MYC for cell proliferation	◦ Requires precise control of culture conditions ◦ Limited application in large-scale production	[67]
Tet-ON/OFF	◦ Reversible, safer, and more controllable immortalization ◦ In the Tet-OFF system, Tet downregulates gene expression ◦ In the Tet-ON system, Tet activates gene expression	◦ Requires precise control of culture conditions ◦ Limited application in large-scale production	[68]
Cre-LoxP	◦ Reversible, safer, and more controllable immortalization ◦ A transgene flanked by LoxP sites is active until Cre recombinase is added when the flanked DNA is excised	◦ Requires advanced expertise and infrastructure. ◦ Potential for unforeseen genomic instability.	[69]

However, if it is compared with the other immortalizing genes, such as viral oncogenes, the immortalizing efficacy of hTERT might not be always as effective as them, depending on the type of cells and maybe donors. Some reports suggested that hTERT alone significantly extends the lifespan of primary cells such as human epithelial cells, endothelial cells, fibroblasts, and MSCs [70,71]. However, when the same cells, such as adipose stromal cells and bone-marrow-derived MSCs, were used for immortalization with hTERT, some reports concluded that hTERT alone was sufficient to immortalize them and extend their lifespans [72,73], but others failed to immortalize them and extend their lifespans, and additional molecular alterations, such as inactivation of p16^INK4a^/pRb and/or p53/p21^WAF1/CIP1^ pathways, are necessary [74,75]. The detailed differences between them have not been clarified but may be dependent on the donor’s heterogeneity and preparation methods and conditions, and so on. In addition, it is well known that oxidative damage to DNA induces cellularly senescent MSCs in culture [76]. Telomerase was reported to have an ability to reduce reactive oxygen species generation and consequently decrease oxidative DNA damage [77,78]. Consistent with these reports, hTERT-immortalized MSCs derived from human adipose tissue showed higher activities of the superoxide dismutase and catalase and significant resistance to oxidative DNA damage than control MSCs [79].

#### 3.1.2. SV40

The SV40 viral oncogenes, particularly the LT and Small T antigen, play a crucial role in the immortalization of host cells [47]. SV40 LT interacts with and inactivates key tumor suppressor proteins p53 and pRb (Figure 5) [48]. By binding to these proteins, SV40 LT disrupts their normal functions and prevents p53 from inducing cell cycle arrest or apoptosis in response to cellular stress or DNA damage, allowing the infected cells to survive and proliferate [49]. By inactivating pRb, LT promotes the transition from the G1 phase to the S phase of the cell cycle, facilitating continuous cell division and growth [50]. ST also contributes to this process, primarily by disrupting the function of pRb and enhancing the effects of LT. It can further promote cell proliferation by altering signaling pathways that regulate cell growth. Together, these proteins effectively bypass the cellular senescence.

#### 3.1.3. HPV E6 and E7

Oncoproteins from HPV, such as E6 and E7, can inhibit tumor suppressor pathways like p53 and pRb, promoting cell cycle progression and extended proliferation (Figure 5) [51]. While this method can successfully immortalize MSCs, concerns about potential tumorigenicity must be addressed through safety checks. HPV, particularly types 16 and 18, is a double-stranded DNA virus that can lead to cervical cancer and other malignancies. E6 and E7 oncoproteins from HPV are commonly used because they are well-characterized for their ability to induce immortalization. E6 and E7 proteins play key roles in the viral life cycle and cellular transformation [52]. E6 protein activates telomerase, promoting cellular immortality by extending telomeres, which normally shorten with each cell division. E6 protein degrades p53 through the proteasome pathway, effectively inhibiting the cell’s ability to respond to DNA damage and inducing apoptosis [53]. This allows for infected cells to continue dividing despite genomic instability. E7 protein binds to and inactivates the pRb [54]. This interaction prevents pRb from binding to the transcription factor E2F, which is crucial for regulating the transition from the G1 phase to the S phase of the cell cycle. As a result, E2F is free to promote the expression of genes necessary for cell cycle progression. The combined effects of E6 and E7 lead to unchecked cellular proliferation and the potential for tumorigenesis. Therefore, the introduction of these viral oncogenes can establish immortalized cell lines.

#### 3.1.4. B Cell-Specific Moloney Murine Leukemia Virus Integration Site 1 (BMI1)

BMI1 is a member of the polycomb group of proteins and engages in diverse cellular processes, including proliferation, differentiation, senescence, and stem cell renewal [55]. In addition, BMI1, as a cancer stem-cell marker, participates in tumorigenesis through various pathways. BMI1 is recognized as a key oncogene that cooperates with c-MYC in promoting tumorigenesis (Figure 5) [56]. p16^INK4a^ is a tumor suppressor that inhibits cyclin-dependent kinases, leading to cell cycle arrest and prevention of uncontrolled cell proliferation. It plays a critical role in cellular senescence and tumor suppression. BMI1 inhibits the expression of p16^INK4a^, thereby blocking the senescence pathway [57]. This allows cells to bypass the growth-inhibitory signals typically activated by p16^INK4a^, promoting continued proliferation. In addition to its role in inhibiting p16^INK4a^, BMI1 also enhances the expression of hTERT [58], which is crucial for maintaining telomere length and enabling cells to divide indefinitely. Through these mechanisms, BMI1 contributes to cellular immortality and tumorigenesis, highlighting its significance in cancer biology and potential as a therapeutic target.

#### 3.1.5. c-MYC Gene Expression

The MYC family of oncogenes, which includes c-MYC, N-MYC, L-MYC, and B-MYC, plays a crucial role in cellular proliferation, differentiation, and immortalization [59]. c-MYC is involved in promoting DNA damage through its regulation of various genes, which can lead to genomic instability. c-MYC blocks the pro-apoptotic activity of p53, allowing cells to evade programmed cell death and continue proliferating even in the presence of DNA damage (Figure 5) [60]. In human prostate epithelial cells, c-MYC maintains telomere length by promoting the overexpression of hTERT, thereby contributing to cellular immortality [61]. This helps prevent the senescence that typically occurs due to telomere shortening during cell division. Overall, the MYC family of genes is integral to the processes that enable cells to bypass normal regulatory mechanisms and achieve unlimited growth, making them significant players in cancer development and progression.

### 3.2. CRISPR/Cas9 Genome-Editing for Immortalization of MSCs

The CRISPR/Cas9 genome-editing system can be utilized for the immortalization of MSCs by targeting specific genes that regulate cellular senescence, cell cycle checkpoints, and telomere maintenance [62]. This approach allows for precise and specific gene targeting, reducing off-target effects compared to viral methods [63,64]. By selectively editing key genes like p53, p16^INK4a^, or hTERT, researchers can control the degree of immortalization, balancing between enhanced proliferation and safety [65]. CRISPR/Cas9 can be used to knock out or modify genes, including tumor suppressor genes like p53. By disrupting p53, which plays a crucial role in regulating the cell cycle and apoptosis, researchers can promote cellular proliferation and bypass senescence, thereby aiding in the establishment of immortalized cell lines [65]. Multiple genes can be targeted simultaneously, enabling a combined approach to tackle different pathways involved in senescence and cell cycle control. Unlike viral methods that integrate oncogenes with higher risks of malignancy, CRISPR/Cas9 provides a more regulated approach with reduced chances of uncontrolled transformation. Despite the precision of CRISPR/Cas9, knocking out tumor suppressor genes like p53 may cause off-target mutations that could have unintended consequences and still increase the risk of cancer formation. Therefore, cells need to be thoroughly tested for signs of transformation before clinical use.

### 3.3. Reversible Immortalization of MSCs

Reversible immortalization of MSCs refers to the temporary induction of immortalization in MSCs, allowing them to proliferate for extended periods while retaining the ability to revert to a non-immortalized, normal state when needed [35,80]. This strategy helps overcome the challenges of limited MSC expansion in culture for therapeutic applications while reducing the risks associated with permanent immortalization, such as tumorigenicity. Genes such as hTERT and SV40 LT, or oncoproteins like HPV E6 and E7 are introduced into MSCs to bypass senescence [69,81]. These genes allow for MSCs to proliferate indefinitely. To make the immortalization reversible, these immortalizing genes are placed under the control of inducible promoters or gene excision systems in temperature-, tamoxifen (TAM)-, tetracycline (Tet)-dependent manors, and site-specific recombination. The mutant of SV40 LT, tsA58, is a temperature-sensitive switch that is active and maintains cell division at 33 °C but inactivated at 39 °C [66]. c-MYCER^TAM^ is a fusion protein of a human c-MYC and a modified mouse estradiol receptor (ER) [67]. In the presence of 4-hydroxytamoxifen (4-OHT), c-MYCER^TAM^ is inactive in the cell cytosol. However, addition of 4-OHT specifically binds to the ER, resulting in c-MYCER^TAM^ dimerization and translocation to the cell nucleus where c-MYC is active as a transcription factor for maintaining cell proliferation. The withdrawal of 4-OHT results in inactivation of c-MYCER^TAM^.

Tet-ON and -OFF systems use Tet-inducible and Tet-repressible elements, respectively. These systems comprise two components; a transcriptional activator of a transcription factor fused to TetR and a transcriptional regulator of a Tet promoter fused to the tetO sequence, respectively [68]. In the Tet-OFF system, in the absence of Tet, the transactivator binds to the tetO element, activating gene expression, while the addition of Tet induces downregulation of gene expression. In the Tet-ON system, in the presence of Tet, the reverse transactivator binds to the tetO element, and the addition of Tet activates gene expression. Cre-LoxP system utilizes a transgene flanked by LoxP sites, and the transgene is active until Cre recombinase is added when the flanked DNA is excised [69].

### 3.4. Suicide Gene Inducible Caspase-9 (iCasp9)

**Figure 6:**
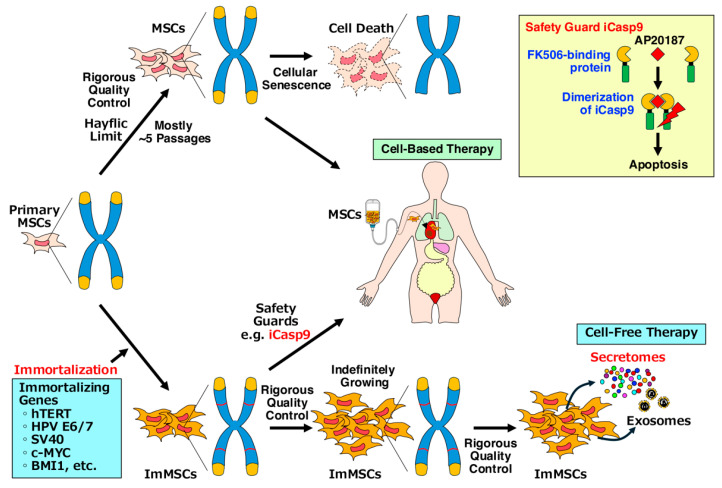
Prolonged culture of primary MSCs and ImMSCs could potentially lead to unwanted genetic changes, which could affect the safety and efficacy of MSC-based therapies. Careful control of their expansion, rigorous safety testing, introducing safeguard system (e.g., iCasp9), and development of alternative strategies like cell-free therapies using only the conditioned medium, secretome, are critical steps to ensure the safe use of MSCs in clinical applications. The iCasp9 system involves a modified version of the Caspase-9 protein that can be activated by a specific small molecule, such as AP1903 and AP20187. When this molecule is administered, it induces the dimerization of the iCasp9 protein, leading to the activation of the apoptotic pathway and subsequent cell apoptosis. This approach allows for the rapid and controlled elimination of MSCs as a safety guard system if any adverse effects arise after transplantation.

The iCasp9 system is a promising strategy for enhancing the safety of MSC transplants [82]. The iCasp9 system involves a modified version of the Caspase-9 protein that can be activated by a specific small molecule, such as AP1903 and AP20187 (Figure 6). When this molecule is administered, it induces the dimerization of the iCasp9 protein, leading to the activation of the apoptotic pathway and subsequent cell death [83]. This approach allows for the rapid and controlled elimination of MSCs if any adverse effects arise after transplantation [84]. This feature is particularly valuable in situations where there is a risk of tumorigenesis or unwanted immune responses, providing a safeguard against them. The ability to induce apoptosis selectively and swiftly enhances the safety profile of MSC therapies. If engineered MSCs show signs of uncontrolled proliferation or tumor formation, they can be quickly eliminated. Monitoring for any unintended consequences of cell elimination and ensuring precise control over the activation of iCasp9 will be crucial for its successful integration into clinical practice.

### 3.5. Combined Methods for Immortalization of MSCs

In the context of cell immortalization, several studies indicate that certain combinations of genetic modifications may be necessary to achieve successful immortalization, because there are several pros and cons of immortalization techniques for MSCs (Table 1). These findings highlight the importance of understanding the specific pathways involved in cell cycle regulation and senescence (Figure 5). By strategically targeting p53 and enhancing telomerase activity, researchers can create more stable and versatile cell lines for various applications, including drug testing and regenerative medicine. However, it is crucial to monitor potential tumorigenic risks associated with such modifications, ensuring that the benefits of immortalization do not come at the cost of safety. Combining different methods for the immortalization of MSCs can be an effective approach to enhance their proliferative capacity while addressing safety concerns, such as tumorigenicity. This hybrid strategy typically involves integrating genetic, epigenetic, and regulatory methods that allow for both efficient immortalization and controlled reversal. The combination of immortalization methods for MSCs leverages the strengths of various genetic and molecular tools to enable efficient and controlled cell expansion. By incorporating mechanisms like inducible systems, epigenetic control, and safety genes such as iCasp9, researchers can immortalize MSCs for therapeutic applications while minimizing the risks associated with long-term culture, such as tumorigenicity. These strategies pave the way for the large-scale production of MSCs, allowing them to be used in regenerative medicine, drug delivery, and disease modeling.

The most widely used immortalizing gene is hTERT, which extends the lifespan of MSCs by maintaining telomere length, preventing replicative senescence. However, the immortalizing potency of hTERT might not be always as good as other viral oncogenes depending on maybe the type of cells and donors, and introduction of hTERT into cells does not always result in their immortalization and extension of lifespan. The other immortalizing genes, such as viral oncogenes, mainly target p53 and pRb pathways, promoting cell cycle progression and bypassing growth arrest. Therefore, the most commonly used combination method is to utilize hTERT and viral oncogenes. Because they utilize these different mechanisms to induce immortalization, the combination of them could lead to cooperative or synergistic effects by increasing the immortalizing efficiency and extending their lifespan. The combination of hTERT and SV40 LT efficiently immortalized human adipose-derived stromal cells. However, the combination reduced differentiation ability and induced chromosomal aberrations and abnormal karyotype, and none of the immortalized cell lines formed tumors in NOD/SCIDγ mice [24,74,75]. In contrast, the combination of BMI1 and hTERT immortalized human adipose-derived stromal cells without any alternations of phenotype and biological activity [24,85].

The multiply-combined method using hTERT, HPV E6 and E7, and BMI1 is a multi-faceted approach that targets key mechanisms, regulating cellular senescence and cell cycle control. By employing these factors together, MSCs can be made to proliferate indefinitely while maintaining their differentiation potential and avoiding senescence. Each of these components contributes uniquely to the immortalization process, enhancing the likelihood of sustained growth with fewer side effects compared to using any single factor alone. If the number of genes used for immortalization increases, the efficiency of immortalization and the ability to extend the lifespan may improve. However, this could also increase the risk of genetic instability and potential transformation during prolonged culture. Alternatively, the combination of multiple genes might compensate for individual tendencies to induce chromosomal instability. The combination of hTERT and HPV E6 and E7 is efficient to immortalize MSCs and extend their lifespan by maintaining telomere length and inactivating p16^INK4a^/pRb and/or p53/p21^WAF1/CIP1^ pathways, respectively. Some papers reported that these immortalized MSCs have no chromosomal abnormalities [75], but other papers suggested that prolonged culture leads to chromosomal instability [86]. Furthermore, the immortalized dental pulp stem cells from human exfoliated deciduous teeth were prepared by introducing all the immortalizing genes of hTERT, HPV E6 and E7, and BMI1 and showed much more increased proliferative activity and cytokine production compared to control primary cells [87]. Thus, although multiple introductions of immortalizing genes may enhance the efficiency of establishing the ImMSCs, rigorously monitoring the chromosomal abnormalities and tumorigenicity is necessary.

## 4. Cell-Based Therapy of ImMSCs

### 4.1. Advantages of ImMSCs in Cell-Based Therapy

Because immortalization provides MSCs with indefinite replication ability, overcoming the natural senescence that limits the expansion of primary MSCs in culture, ImMSCs have been increasingly utilized in regenerative medicine [21,22,40,88]. Immortalization is achieved through genetic modification, typically by introducing genes such as hTERT, SV40, HPV E6 and E7, BMI1, and c-MYC that extend the replicative lifespan of MSCs while maintaining their functionality. These ImMSCs can be used in various regenerative and therapeutic applications, owing to their capacity for self-renewal, multipotency, and secretion of bioactive molecules that facilitate tissue repair, immunomodulation, and anti-inflammatory effects. ImMSCs can proliferate indefinitely, allowing for the production of sufficient quantities of MSCs. Immortalization of MSCs overcomes the limitations by enabling the continuous expansion of cells, providing a reliable source for therapeutic use. Moreover, using the same immortalized cell line can help overcome the issue of donor heterogeneity, ensuring more consistent quality in MSC-based therapies. Immortalized MSC lines offer batch-to-batch consistency, which is important for clinical and commercial applications. Since ImMSCs do not require repeated isolation from donors, they reduce the cost and time involved in producing therapeutic cell products.

### 4.2. Safety Considerations of ImMSCs in Cell-Based Therapy

ImMSCs are genetically modified, which introduces a risk of tumorigenicity. The combination of long-term proliferation potential and genetic alterations, such as overexpression of hTERT or inhibition of p53 or pRb, increases the chance of malignant transformation. To mitigate this, tumorigenicity assays, including karyotyping analyses or soft agar colony formation, must be performed to ensure that ImMSCs do not form tumors. Additionally, inducible suicide genes such as iCasp9 can be incorporated as a safety mechanism to eliminate the cells if they show signs of malignancy [82,83]. While MSCs have immunomodulatory properties and are considered to be immunoprivileged, expressing low levels of MHC class II and costimulatory molecules, ImMSCs may be recognized as foreign by the host immune system and acquire new antigens or aberrant expression of proteins that could provoke an immune response. To address this, immune tolerance of ImMSCs should be evaluated, and steps can be taken to ensure they are safe for clinical use, such as modifying cell surface markers. Continuous monitoring of the genetic stability of ImMSCs during long-term culture is critical. Regular testing for chromosomal abnormalities, genetic mutations, and changes in cell properties is necessary to ensure the safety of the cells for therapeutic applications.

### 4.3. Therapeutic Applications of ImMSCs in Cell-Based Therapy

Cell-based therapies using MSCs have shown potent therapeutic effects against a variety of diseases. In place of primary MSCs, several studies also demonstrated that cell-based therapy using ImMSCs have a promising therapeutic effect as an unlimited source of MSCs to supply continuously [21,40,88]. Increased proliferative activity and secretion ability of ImMSCs due to immortalization would potentiate their therapeutic effects.

For instance, although MSCs have shown therapeutic effects against transient cerebral ischemia, similar therapeutic effects, such as reduced lesion volume and functional improvement, were reported with intravenous infusion of hTERT-ImMSCs derived from bone marrow in a rat cerebral ischemia–reperfusion model [89]. When MSCs and hTERT-ImMSCs were compared, hTERT-ImMSC transplantation showed better therapeutic effects, including alleviated brain edema and cerebral infarction, decreased apoptosis of brain cells, and improved neural function than MSC transplantation [90]. hTERT-ImMSCs derived from bone marrow named YKNK-12 showed much increased growth properties and possessed multipotency [91]. The osteogenically differentiated YKNK-12 cells produced factors, which have promoting ability in bone formation, such as bone morphogenetic protein (BMP)4, BMP6, fibroblast growth factor (FGF)6, FGF7, transforming growth factor (TGF)-β1, and TGF-β3. Transplantation of these differentiated cells into calvarial bone defect model mice showed profound calvarial bone healing with enhanced expression of human osteocalcin, runt-related transcription factor 2, alkaline phosphatase, and osterix in the bone-regenerating area. Of note, the mice transplanted with YKNK-12 cells showed no development of tumors for at least 6 months.

One of the most promising immortalized cell lines for translating into practical medicines is the reversibly immortalized human fetal NSC line CTX0E03 (ReNeuron) [67]. Compared to MSCs, NSCs are considered to be more suitable because NSCs have much potent capacity for engraftment and neural cell differentiation. CTX0E03 is an immortalized NSC line with c-MYCER^TAM^, which is c-MYC gene fused with a modified mouse ER. The activity of the c-MYCER^TAM^ protein is present as an inactive monomer in the cytosol of cells. By adding 4-OHT, the c-MYCER^TAM^ protein forms a dimer and translocates to the nucleus as a transcription factor, resulting in the establishment of an increased and stable proliferating cell line. Implantation of CTX0E03 cells in a rat model of stroke caused by transient middle cerebral artery occlusion showed improved sensorimotor dysfunctions and motor deficits in the site of implantation [92]. In a phase 1, first-in-man study, single intracerebral doses of CTX0E03 up to 20 million cells induced no adverse events and improved neurological function at two years post-implantation [93].

## 5. Cell-Free Therapy of Secretome of ImMSCs

### 5.1. Advantages of Secretome of ImMSCs in Cell-Free Therapy

Secretome refers to the conditioned medium or culture medium collected from ImMSCs after they have been cultured for a certain period in a serum-free medium [15,94,95,96]. It is a mixture enriched with a variety of bioactive molecules secreted by ImMSCs themselves. The secretome includes cytokines, which modulate the immune system and reduce inflammation; growth factors, which promote tissue repair and regeneration; and exosomes [15,94,95,96]. Exosomes are small extracellular vesicles, ranging from 30 to 150 nm in diameter, secreted by various cell types, including MSCs [97]. These vesicles play a significant role in intercellular communication by transferring bioactive molecules such as proteins, lipids, mRNAs, and microRNAs to recipient cells, influencing their function. The secretome is a pure production from only ImMSCs without exogenously adding any other recombinant growth factors in the medium.

Migratory and multipotential abilities of MSCs likely well account for possibility of cell replacement where damaged tissue could be repaired. However, the concept has shifted to paracrine effects by MSCs’ ability to produce cytokines, growth factors, and exosomes that stimulate tissue repair and regeneration and immunomodulation [6]. Immortalizing MSCs, typically through methods such as hTERT expression, provides a continuous and stable source of these secreted factors, overcoming limitations such as the short lifespan and senescence of primary MSCs. Since the secretome contains no live cells, the risk of immune rejection is minimized, even in allogeneic settings, and concerns regarding tumorigenesis, or transmission of infections are reduced. ImMSCs can proliferate indefinitely, allowing for mass production of secretomes with consistent quality and potency. This is particularly important for developing off-the-shelf therapeutic products. MSC secretome production may be less expensive than direct MSC therapies due to streamlined manufacturing processes and reduced infrastructure needs. Secretomes can be administered in various ways, including intravenous, intranasal, and topical ways, and allows for easier handling, storage, and delivery.

### 5.2. Safety Considerations of Secretome of ImMSCs in Cell-Free Therapy

The use of ImMSCs in cell-free therapies presents both opportunities and challenges, particularly regarding tumorigenicity. By using the bioactive factors secreted by ImMSCs, the risk of tumor formation or other complications is significantly reduced compared to administering immortalized cells directly. Secretomes are less likely to trigger immune rejection, making them a safer option for allogeneic therapy. However, in the case of exosome regenerative therapy, there are concerns about the potential for uncontrollable transfer of genetic information from one cell to another, which could lead to unintended consequences, including promotion of tumorigenesis in recipient cells [98]. Although transformed ImMSCs do not directly release cancer cells into the secretome (conditioned media), the quality and composition of the secretome can change due to genetic instability during prolonged culture depending on the culture conditions. This may affect the therapeutic properties and safety of the derived products. There is a risk that altered secretomes could include factors that promote cell proliferation or survival, potentially leading to unintended consequences when administered to patients. It is essential to perform extensive characterization of ImMSCs and their secretomes to assess any changes in their biological properties. Implementing strict quality control measures throughout the production process can help minimize risks associated with the use of ImMSCs in therapy.

### 5.3. Therapeutic Applications of Secretome of ImMSCs in Cell-Free Therapy

Because ImMSCs stably and consistently secretes more factors, including cytokine, growth factors, and exosomes, than MSCs; these cells are ideal sources for collection of secretomes. Generally, secretomes from MSCs and even ImMSCs do not contain any harmful materials to induce tumorigenesis. Similar to the cell-based therapy using ImMSCs, cell-free therapy using the seretome has shown therapeutic effects against a variety of diseases, including degenerative nerve and bone, autoimmune, and inflammatory diseases [21]. Several examples are shown below.

For instance, several secretomes isolated from human adipose-derived MSCs immortalized by hTERT were reported to be beneficial in skin regeneration and wound healing. Human adipose tissue MSC lines (HATMSCs) immortalized by hTERT secreted a wide range of cytokines associated with angiogenesis, such as angiogenin, growth-regulated oncogene, IL-6, IL-8, vascular endothelial growth factor, insulin growth factor 1, and matrix metalloproteinase. The supernatant enhanced the survival of fibroblasts, endothelial cells, and keratinocytes in a chronic wound model and showed more strong fibroblast proliferation than coculture with fibroblast and HATMSCs [99]. The production of these cytokines was higher than that from primary MSCs, and the pre-conditioning by stimulation in hypoxia or with immunomodulatory factors during culture augmented the different cytokine production patterns [100]. Secretomes of human adipose-derived mesenchymal stromal cells immortalized by hTERT induced the proliferation and migration of keratinocytes, dermal fibroblasts, and endothelial cells [101]. They sustained the complete re-epithelialization of a full-thickness wound performed on a skin organotypic model. Secretomes of human adipose-derived mMSCs immortalized by hTERT (ASC52telo) interfered with fibrogenesis using a model of TGF-β-induced differentiation of fibroblasts into myofibroblasts [102]. In the secretomes, fibrosis-associated microRNAs, including microRNA-21 and microRNA-29c, mediated the anti-fibrotic effects.

Exosomes were isolated from human fetal NSCs and ESC-derived MSCs and immortalized by c-MYC. Cell-based therapy using the reversibly c-MYCER^TAM^-immortalized human fetal NSC line CTX0E03 has been shown to be beneficial in ischemia–reperfusion injury in the ischemic stroke [103]. Similar protective effects on the heart from ischemia–reperfusion injury by the exosomes isolated from the conditioned medium of differentiating CTX0E03 cells were reported [104]. Exosomes were isolated from human ES cell-derived MSCs immortalized by c-MYC were shown to reduce relative infarct size in a myocardial ischemia–reperfusion model mice [105]. In addition, topical application of these exosomes in an imiquimod-induced psoriasis-like inflammation inhibited complement activation, specially C5b-9 complex formation through CD59 in the skin stratum corneum and thereby alleviated IL-17 release by neutrophil extracellular traps from neutrophils that accumulate in and beneath the stratum corneum [106].

Human exfoliated deciduous teeth are a unique source of stem cells, which are developed embryologically from the neural crest and therefore have great potential of differentiating into ectodermal lineages such as neurons and glia cells [107,108]. However, the number of stem cells obtained from a human exfoliated deciduous tooth is very low, compared to that of the other MSC sources such as bone marrow, adipose tissue, and umbilical cord. Therefore, immortalization of dental pulp stem cells from human exfoliated deciduous teeth is very useful for the generation of good cell sources for producing secretomes effective for tissue repair and regeneration of nerve cells. Indeed, the secretome of dental pulp stem cells from human exfoliated deciduous teeth immortalized by hTERT, HPV E6 and E7, and BMI1 contain a variety of cytokines and growth factors and showed therapeutic effects on the pressure ulcer formation in a cutaneous ischemia–reperfusion mouse model established using two magnetic plates [87]. Antioxidative and angiogenic activities mediated by hepatocyte growth factor and vascular endothelial growth factor in the secretome contributed to alleviation of the pressure ulcer formation.

## 6. Tumorigenicity

### 6.1. Tumorigenesis

Tumorigenesis, also known as oncogenesis or carcinogenesis, is the process through which normal cells transform into cancerous cells, ultimately leading to the development of a tumor (Figure 3). This process involves a complex series of genetic, epigenetic, and environmental changes that result in uncontrolled cell growth, resistance to cell death, and the ability to invade other tissues. Tumorigenesis consists of three key stages: initiation, promotion, and progression [109]. The initiation stage involves genetic mutations or damage to DNA that can arise due to carcinogens such as tobacco smoke, radiation, and chemicals, viral infections, or inherited genetic defects. These mutations typically affect oncogenes, tumor suppressor genes, and DNA repair genes [110]. Mutations in proto-oncogenes (e.g., RAS, MYC, epidermal growth factor receptor) convert them into oncogenes, which drive uncontrolled cell growth. Loss or inactivation of tumor suppressor genes [e.g., p53, pRb, breast cancer susceptibility gene (BRCA)1, adenomatous polyposis coli] removes critical regulatory checkpoints, leading to unchecked proliferation (Figure 5) [111]. Defects in DNA repair mechanisms, such as BRCA1/BRCA2 mutations, increase the accumulation of mutations, accelerating the progression toward malignancy. Cells with DNA mutations can either repair the damage or undergo apoptosis by programmed cell death. However, if these mechanisms fail, the damaged cells may survive and proliferate. In the promotion stage, proliferative signals allow for mutated cells to continue dividing. Although not cancerous yet, these cells have an abnormal rate of growth. Mutations in genes that control cell division, like p53 or pRb, prevent the normal regulation of the cell cycle, allowing cells to bypass growth arrest and apoptosis. Inflammation or exposure to certain hormones or growth factors can also promote the survival and proliferation of these mutated cells. Cancer cells develop mechanisms to avoid apoptosis, the programmed cell death pathway that eliminates damaged or abnormal cells, through B-cell CLL/lymphoma-2 or surviving. 

Cells in the progression stage acquire further mutations that enhance their malignant behavior, including the ability to grow in an uncontrolled manner and resist cell death (Figure 3) [109]. Genetic instability allows cells to accumulate more mutations, leading to aggressive cancer traits. At this point, cells may also develop the ability to stimulate blood vessel growth via angiogenesis, enabling the tumor to receive nutrients and oxygen. The growth of new blood vessels from existing vasculature is essential for tumor growth beyond a small size, as tumors need a blood supply to bring oxygen and nutrients. Vascular endothelial growth factor is one of the key drivers of the angiogenesis in tumors. Cancer cells often activate telomerase or other mechanisms to maintain their telomeres, allowing them to bypass the usual limits on cell division. This transformation stage marks the transition to a malignant tumor, which is characterized by invasion into surrounding tissues and, potentially, metastasis spreading to distant organs. Tumor cells eventually acquire the ability to invade surrounding tissues and spread to distant organs via the blood or lymphatic system, a process called metastasis. Metastatic cells exhibit changes in cell adhesion molecules like E-cadherin, allowing them to detach from the primary tumor and migrate to other sites. This biological process is called epithelial–mesenchymal transition [112], where epithelial cells that are usually organized and tightly connected lose their characteristics and acquire the properties of mesenchymal cells, which are more migratory and invasive. This transition plays a key contributor to tumor progression, metastasis, and fibrosis in pathological conditions and embryonic development, wound healing, and tissue regeneration in physiological processes. Tumor cells often evade detection by the immune system by downregulating major histocompatibility complex molecules, secreting immunosuppressive factors, or recruiting regulatory immune cells like regulatory T cells that suppress anti-tumor immunity.

### 6.2. Potential Tumorigenicity of MSCs

The stem cell hypothesis of cancer development is also known as the cancer stem cell (CSC) hypothesis, suggesting that tumors are driven by a small subset of cells within the tumor that have stem-cell-like properties [113,114,115,116]. These CSCs are capable of self-renewal, differentiation, and tumor initiation, much like normal stem cells in healthy tissues. While MSCs are considered safer than pluripotent stem cells in terms of tumor formation, they are not completely free of risks. MSCs possess inherent homing abilities, allowing them to migrate to the sites of injury or inflammation, including tumors [117,118]. This property makes them ideal candidates for targeted drug delivery to cancers. MSCs can secrete a variety of bioactive factors, such as cytokines, growth factors, and extracellular vesicles, which can modulate the tumor microenvironment. This secretion can affect the phenotype of cancer cells, potentially promoting the progression of cancers or inhibiting it.

Under certain conditions, MSCs have the potential to transform and develop into CSCs, which can contribute to tumor growth [119,120,121,122]. Prolonged in vitro expansion of MSCs can lead to chromosomal instability and consequent genetic alterations may accumulate, increasing the risk of malignant transformation. These transformed MSCs can potentially acquire characteristics of cancer cells, including uncontrolled proliferation and resistance to apoptosis. In some studies, MSCs have been implicated in contributing to cancer progression by differentiating into cancer-associated fibroblasts, which can enhance tumor growth by secreting factors that promote angiogenesis and suppress immune responses [123,124]. In addition, MSC-derived exosomes have been shown to affect the tumor microenvironment by promoting metastasis and tumorigenic signaling [97,125,126]. When MSCs are introduced into a cancerous microenvironment, they may undergo transformation into cancer stem-like cells due to the influence of the cytokines, growth factors and exosomes present in the tumor microenvironment. This interaction can enhance the potential for tumor progression. MSCs can be hijacked by tumors, acquiring characteristics of CSCs. This transformation is linked to pathways involving p53 inactivation, WNT signaling, and TGF-β signaling, leading to enhanced tumorigenicity [127,128]. Several studies have raised concerns about the long-term safety of MSC therapies, especially with respect to the risk of tumor formation. Preclinical models have shown that transformed MSCs can form sarcoma-like tumors [129]. Although MSCs show immense promise for regenerative medicine, their potential to undergo malignant transformation and contribute to tumor formation is a real concern. Careful control of their expansion, rigorous safety testing, introducing safeguard system, and development of alternative strategies like cell-free therapies using only the conditioned medium, secretome, are critical steps to ensure the safe use of MSCs in clinical applications (Figure 5).

### 6.3. Genetical Stability of MSCs in Culture

The clinical application of MSCs requires a large number of cells, and due to the naturally low number of MSCs found in vivo, extensive ex vivo expansion is necessary. However, such expansion carries the risk of introducing genetic changes. Prolonged cell proliferation in vitro can lead to the accumulation of mutations and chromosomal aberrations, potentially resulting in malignant transformation. Therefore, it is critical to conduct cytogenetic and molecular analyses to ensure that the MSCs used for cell-based therapy are appropriate and safe. While MSCs have been shown to proliferate continuously in vitro, it remains uncertain how many passages can be performed before the cells begin to acquire chromosomal instability or lose their multipotency. Research using different sources, culture conditions, and passage densities has yielded varied results [130,131,132]. Some studies suggest that during early passages (P1 to P5), MSCs maintain a normal karyotype, while at later passages (P6 and beyond), they may begin to exhibit chromosomal aberrations [133,134,135]. Cells exhibiting clonal aneuploidy, that is, abnormal chromosome and chromosomal translocations, have been reported as early as the first and fourth passages in some MSC lines depending on donors. Other research indicates that MSCs cultured in α-MEM medium with 20% fetal bovine serum retain normal karyotypes up to passage 20. Another study found that while aneuploidy can occur in MSCs, it does not persist over extended culture and is not linked to malignant transformation [130]. The disappearance of karyotypically abnormal cells might be due to apoptosis, cell cycle arrest, or replicative senescence caused by harmful genetic mutations. Ultimately, the factors that determine the genetic stability of MSCs during prolonged culture remain unclear, and further investigation is required to fully understand these dynamics. Therefore, in most clinical trials involving MSCs, the cells are used at early passages, typically no more than passage 5.

## 7. Assays for Tumorigenesis

The expansion of MSCs in culture can lead to chromosomal instability, which increases the risk of transformation and tumor formation. To assess chromosomal stability and abnormalities in MSCs, various techniques can be employed as shown below (Figure 3). These methodologies are crucial for monitoring the genetic stability of MSCs and ensuring their safety for therapeutic applications.

### 7.1. Fluorescence In Situ Hybridization (FISH) [136]

FISH is a powerful technique used to detect and localize specific DNA sequences on chromosomes. In FISH, fluorescently labeled DNA probes are designed to bind to particular genes or chromosomal regions of interest. The probes hybridize to their complementary DNA sequences on the chromosomes, allowing for specific targeting. Under a fluorescence microscope, the bound probes emit light, enabling visualization of the location and presence or absence of the targeted sequences. FISH is widely used for various applications, including detecting chromosomal abnormalities, identifying genetic disorders, and studying gene expression patterns. Its ability to provide spatial information about DNA sequences makes it invaluable in both research and clinical settings.

### 7.2. Spectral Karyotyping (SKY) [137]

SKY is an advanced technique that enhances traditional FISH by using chromosome-specific fluorescent labels to visualize all chromosomes in a single hybridization. Each chromosome is labeled with a unique fluorescent dye, allowing all 24 chromosomes to be displayed in different colors simultaneously. SKY can detect chromosomal abnormalities without needing prior knowledge of the specific abnormalities present, making it a powerful tool for comprehensive genomic analysis. The use of multiple colors allows for clear differentiation between chromosomes, facilitating the identification of structural rearrangements, aneuploidies, and other chromosomal abnormalities. SKY is useful in cancer research and diagnostics, as it provides a detailed overview of chromosomal changes that may be associated with tumorigenesis.

### 7.3. G-Banded Karyotyping [138]

Karyotyping is a vital technique used to analyze the chromosomal composition of cells, providing insights into genetic abnormalities and the overall chromosomal architecture of an organism. The karyotype refers to the complete set of chromosomes in a species or an individual, characterized by their size, number, and shape. Karyotyping process involves preparing metaphase spreads of chromosomes, staining them, and then examining them under a microscope to determine the chromosome complement of an individual. This includes counting the total number of chromosomes and identifying any structural or numerical abnormalities. This G-banded karyotyping-specific method uses Giemsa staining to produce a distinct banding pattern on chromosomes, which helps detect various chromosomal abnormalities, including deletions, duplications, translocations, and inversions. Karyotyping is widely used in clinical genetics, cancer research, and prenatal screening to identify chromosomal abnormalities associated with various diseases and conditions.

### 7.4. Array Comparative Genomic Hybridization (aCGH) [139]

aCGH is a powerful genomic tool used to evaluate DNA copy number variations associated with chromosomal abnormalities. aCGH is designed to detect genomic imbalances, such as deletions and duplications, which may not be visible through traditional G-banded karyotyping. One of the major advantages of aCGH is its ability to identify smaller and more subtle DNA copy number variations than those typically detected by G-banding. This allows for a more detailed analysis of the genome. In aCGH, genomic DNA from the test sample is labeled and hybridized to a microarray containing probes that represent various genomic regions. The relative fluorescence intensity of the probes indicates the presence or absence of DNA segments, allowing for a comparison between the test and control samples. Thus, aCGH provides a high-resolution overview of the genome and is widely used in clinical settings for diagnosing genetic disorders, identifying cancer-related chromosomal abnormalities, and exploring genomic variations in research settings.

### 7.5. Single Nucleotide Polymorphism (SNP) Arrays [140]

SNP is a common type of genetic variation where a single nucleotide in the DNA sequence is altered at a specific position in the genome. SNPs are the most prevalent type of genetic variation in humans and occur approximately once every 300 nucleotides, making them abundant throughout the genome. Due to their frequency and stability, SNPs are valuable as biomarkers in various fields. SNPs can be linked to susceptibility to certain diseases or conditions. They can influence individual responses to drugs, helping tailor personalized medicine approaches. SNPs are used in genome-wide association studies to identify genetic variants associated with complex traits.

### 7.6. Next-Generation Sequencing (NGS) [141]

NGS is a powerful technology that allows for the rapid sequencing of DNA or RNA, providing comprehensive insights into genetic variation and expression. NGS can sequence millions of fragments simultaneously, allowing for large-scale genomic studies. Compared to traditional sequencing methods, NGS is faster and significantly less expensive, making it accessible for various research applications. The ability to provide comprehensive genetic insights makes it an invaluable tool in both research and clinical diagnostics including SNPs, insertions, deletions, and structural variations associated with diseases and tumor heterogeneity, mutations, and copy number variations in cancer research.

### 7.7. Soft Agar Colony-Forming Assay [142]

Anchorage-independent growth is a critical characteristic of cancerous cells, enabling them to proliferate without the need for attachment to a solid surface, which is a typical requirement for normal cell growth. This ability signifies a shift in cellular behavior associated with malignant transformation. The soft agar colony formation assay is a widely used experimental technique to assess this property of cells. In this assay, cells are embedded in a semi-solid agar medium, which allows for the formation of colonies without the need for attachment to a surface. The agar supports the cells while preventing them from spreading too far. The formation of colonies in soft agar is indicative of cellular transformation and tumorigenic potential. Normal cells typically do not grow well in soft agar, while cancer cells can proliferate and form colonies. The number and size of colonies can be quantified to assess the effects of different treatments. This assay is essential in cancer research for screening potential oncogenic properties of cells, evaluating the effectiveness of anticancer therapies, and studying mechanisms of transformation and metastasis.

### 7.8. Transplantation into Immunodeficient Rodents [84]

Immunodeficient rodents are invaluable models for studying tumorigenicity and cancer biology, particularly because they lack functional immune systems that would otherwise reject transplanted cells or tissues. Followings are an overview of commonly used immunodeficient rodent models [143]:

Nude Mice (BALB/cAJcl-nu/nu): This mouse line lacks a thymus, resulting in a severe deficiency in T-cell function, and is widely used for transplanting human tumors and studying tumor growth.
SCID Mice (C.B-17/Icr-scid/scidJcl): This mouse line has a mutation that prevents the development of functional B and T lymphocytes and is useful for studying human immune responses and tumorigenicity.
NOD-SCID Mice (NOD/ShiJic-scidJcl): This mouse line is generated by combining the SCID mutation with a NOD background, which also has defective innate immunity, allowing for better engraftment of human cells and tumors.
NOG Mice (NOD/Shi-scid, IL-2RγKO Jic): This mouse line lacks T, B, and NK cells, making them even more immunocompromised than NOD-SCID mice, and is ideal for human cell or tissue engraftment studies.
Nude Rats (F344/NJcl-rnu/rnu): Similar to nude mice but in a rat model, this rat line provides alternatives for larger studies.

After transplantation of cells into these rodents, tumor development is typically evaluated several months later. The assessment involves hematoxylin and eosin staining is a standard histological staining technique that allows for visualization of tissue architecture and cellular morphology. Tumors can be assessed for characteristics such as size, structure, cellularity, and any signs of malignancy. These models are essential for evaluating the tumorigenic potential of cell lines or stem cells.

## 8. Challenges in Gaining Regulatory Approval for Genetically Modified ImMSCs

Regulatory issues and challenges in gaining FDA (U.S. Food and Drug Administration), EMA (European Medicines Agency), and PMDA (Pharmaceuticals and Medical Devices Agency in Japan) approval for genetically modified ImMSCs are multifaceted and demand careful consideration of scientific, technical, and ethical aspects. Successfully navigating the regulatory landscapes of them for immortalized MSCs involves addressing scientific uncertainties, demonstrating long-term safety, and complying with region-specific guidelines. Collaborative efforts to harmonize regulations and advance technologies for safer and more effective ImMSC therapies are crucial for expediting approvals and patient access. ImMSCs require stringent manufacturing standards to ensure safety, purity, and consistency. Viral vector safety, addressing risks associated with integrating viral vectors, such as insertional mutagenesis, is crucial. Product consistency that maintains uniform genetic modification across cell batches is critical. Scalability to transit from laboratory-scale production to Good Manufacturing Practice (GMP)-compliant, large-scale production poses technical and logistical hurdles. Regulators also require comprehensive data on preclinical studies, such as not only therapeutic efficacy but also genotoxicity and tumorigenicity risks due to genetic modifications, immunogenicity, off-target effects, and stability of genetic alterations during cell expansion. In addition, challenges include clinical trials to demonstrate both efficacy and long-term safety, addressing patient heterogeneity.

## 9. Future Prospect of ImMSCs and Their Secretomes in Therapeutic Applications

Immortalizing MSCs via methods, such as hTERT expression, or combining with oncoproteins like HPV E6 and E7 or BMI1, enables these cells to bypass senescence and continue proliferating indefinitely. This offers several potential benefits, including scalability, consistency, and extended functional capacity. ImMSCs provide a constant and unlimited cell source for therapeutic applications, reducing the variability and supply issues associated with primary MSCs. They allow for the production of consistent, well-characterized batches, critical for large-scale clinical applications. These cells could potentially offer more robust and longer-lasting therapeutic benefits in tissue repair and regeneration.

ImMSCs and their secretomes could play a major role in personalized medicine by tailoring secretome composition to match individual patient needs for treating specific conditions, such as neurodegenerative diseases, cardiovascular diseases, and immune disorders. Cell-free therapies using the secretome, including exosomes derived from ImMSCs, are gaining more significant attention due to their therapeutic potential without the risks associated with direct cell transplantation. By using the secretome form ImMSCs, the risk of tumor formation or other complications is significantly reduced compared to administering ImMSCs directly. Secretomes are cheaper to produce and store compared to live cell therapies, making them a more feasible option for widespread clinical use. Secretomes are less likely to trigger immune rejection, making them a safer option for allogeneic therapy. Exosomes have the natural ability to deliver their contents, including proteins, miRNAs, and lipids, to specific cells, enhancing therapeutic precision. Using exosomes from ImMSCs deliver personalized therapies based on biomarkers identified in patients’ specific disease profiles. Exosome regenerative therapy represents a cutting-edge approach in the field of regenerative medicine, leveraging the natural properties of exosomes for targeted therapeutic delivery. While their potential benefits are significant, ongoing research is essential to address safety concerns and optimize their use in clinical settings.

One of the primary concerns with ImMSCs is tumorigenicity, especially since immortalization processes of hTERT and oncogene overexpression might increase the risk of malignant transformation. Rigorous safety tests, such as soft agar assays, transplantation into immunodeficient rodents, and monitoring for chromosomal abnormalities using FISH or karyotyping, are critical to ensure safety. Technologies like inducible hTERT or suicide genes, such as iCasp9, may allow for the controlled proliferation of ImMSCs and induce apoptosis after their therapeutic task is completed, minimizing tumorigenic risk. Understanding and addressing the balance between therapeutic potential and tumorigenic risk will be crucial for the successful translation of ImMSC therapies into clinical practice. Bringing ImMSCs and their secretomes into clinical practice will require strict adherence to GMP standards. Quality control including ensuring the purity, stability, and potency of the products will be essential, especially as ImMSCs undergo long-term culture. Large-scale production methods need to be optimized to generate clinical-grade secretomes that maintain consistency across batches.

Thus, to translate MSC-based therapy and cell-free therapy using MSCs into practical medicine, the immortalization of MSCs would be practically inevitable and essential method. The future prospect of ImMSCs and their secretomes in therapeutic applications is highly promising, and they represent an exciting frontier in regenerative medicine, with the potential to provide scalable, cost-effective, and efficient therapies. Further addressing safety concerns, standardizing production methods, and demonstrating consistent therapeutic efficacy are critical steps before these innovations can reach widespread successful clinical translation. Continued research and collaboration among stakeholders will be essential to achieving these goals.

## Data Availability

Not applicable.
